# Reliability and reactivity of heart rate variability and pupillometry in response to controlled autonomic perturbations in university students

**DOI:** 10.3758/s13428-025-02793-1

**Published:** 2025-08-19

**Authors:** Michael Matyevich, Michael I.C. Kingsley, Rodney Bice, Michael Mortimer, Ben Horan, Stefan Piantella, Bradley J. Wright

**Affiliations:** 1 Department of Psychology, Counselling and Therapy, School of Psychology and Public Health, La Trobe University, Melbourne, VIC Australia; 2 Holsworth Research Initiative, La Trobe University, Bundoora, VIC Australia; 3 Department of Exercise Sciences, University of Auckland, Auckland, New Zealand; 4 School of Engineering, Deakin University, Geelong, VIC Australia; 5 Healthy Brain and Mind Research Centre, School of Behavioural and Health Sciences, Australian Catholic University, Fitzroy, VIC Australia

**Keywords:** CONVIRT, Guided breathing, HRV, Minimal detectable change, Stress, Pupil asymmetry

## Abstract

The aim of this test–retest reliability study was to evaluate the reliability and reactivity of heart rate variability (HRV) and pupillometry metrics under conditions with controlled cognitive stimulation and paced breathing within a virtual reality protocol. After habituation, 30 English-speaking university students completed a four-phase protocol on two occasions separated by 1 week. HRV and pupillometry were continuously measured during the following phases: baseline, cognitive testing, guided breathing with nature immersion, and spontaneous breathing with nature immersion. Strong day-to-day relative reliability was confirmed for both HRV (pooled ICC: 0.75 to 0.83) and pupillometry (pooled ICC: 0.66 to 0.87). HRV metrics of sympathovagal balance in the time, frequency, and non-linear domains showed reactivity with significant differences between all phases. Pupillometry metrics increased progressively from cognitive testing to guided breathing nature immersion to nature immersion, suggesting psychological rather than respiratory influences. Relatively large minimal detectable change values were determined across HRV (22 to 54% deviation from baseline) and pupillometry (33 to 88% deviation from baseline) metrics. Although the relatively large ratio limits of agreement and minimal detectable change values suggest that detecting systematic changes in these metrics over time might be difficult at the individual level, strong relative reliability supports the use of HRV and pupillometry metrics to detect differences in sympathovagal balance between groups. Additionally, the responsiveness of these metrics demonstrates the efficacy of the proposed virtual reality protocol in inducing detectable physiological reactivity across HRV and pupillometry metrics.

## Introduction

There is well-established evidence that imbalanced autonomic nervous system (ANS) function, through overactivation of the neurophysiological stress response (Jarczok et al., 2020; Wyller et al., 2009), is implicated in many chronic health conditions (Eddy et al., 2016). For example, autonomic dysfunction has been associated with increased risk of developing cardiovascular disease (Lupien et al., 2009; Merz et al., 2002), myocardial infarction (Bigger Jr et al., 1992), and neurological conditions such as depression (Burns et al., 2014; Hemmerle et al., 2012; Novakova et al., 2013). Autonomic arousal has also been associated with acute decrements in functioning, including decreased cognitive performance and increased anxiety (Corrone et al., 2021; Levy, 2013; Miu et al., 2009; Wright et al., 2022). Therefore, chronic autonomic arousal can have deleterious effects on both long-term health and optimal function.

In addition to chronic change in autonomic balance, the impact of changes in physiological state is detectable through measurement of acute reactivity, whereby chronically stressed individuals have been shown to exhibit greater physiological reactivity to acute conditions (Castaldo et al., 2015; Pike et al., 1997), or blunted responses possibly related to burnout (Al Abdi et al., 2018; Brugnera et al., 2019; Schiweck et al., 2019; Wekenborg et al., 2019; Xin et al., 2020). Therefore, changes in acute physiological responsiveness, measured by ANS reactivity, provide an opportunity to identify those who are at increased risk of poor health outcomes associated with chronic autonomic arousal (Manser et al., 2021). The first step, however, is understanding precisely what constitutes a ‘change’ in autonomic arousal. To do this, it is important to determine ‘normal’ variation among indices of autonomic activity.

The sympathetic nervous system (SNS) initiates a physiological state of autonomic arousal (Vingerhoets, 1985), which is typically dominant during periods of perceived stress (Wirtz & von Känel, 2017). Responses include cardiac output becoming more stable (Pereira et al., 2017) and a predominant state of pupil dilation (Turnbull et al., 2017). Conversely, the parasympathetic nervous system (PNS) exerts influence during periods of rest and low perceived stress (Vingerhoets, 1985), where cardiac output becomes more varied, and pupil constriction occurs (Kim et al., 2022). A more flexible, adaptable ANS function suggests a healthy balance in the interchange between the SNS and PNS (Pereira et al., 2017) and a high level of resilience to the physical and psychological impact of stressful states (McCraty & Shaffer, 2015). Therefore, physiological indices estimating sympathetic and parasympathetic influence, can indicate the physical, emotional, or cognitive state of an individual, particularly in relation to stress (Parnandi & Gutierrez-Osuna, 2013).

Heart rate variability (HRV) is a non-invasive measure of autonomic balance (Malik, 1996; Riganello et al., 2012; Shaffer et al., 2014), where HRV reflects the changes in timed intervals between consecutive heartbeats, also known as R-R intervals (Malik, 1996). Low total HRV can be indicative of SNS predominance, suggesting an overactive stress response (Kim et al., 2018). This state is associated with many poor health conditions including chronic stress (Jarczok et al., 2020; Shaffer et al., 2014), cardiovascular disease (Fang et al., 2020), neurological conditions (Thayer et al., 2012) and mortality (La Rovere et al., 2022). Additionally, higher total HRV has been linked to greater physiological resilience, coping capacity, and adaptability (McCraty & Shaffer, 2015; Shaffer et al., 2014; Thayer et al., 2012). Multiple indices of HRV are used as proxy measures of autonomic arousal. For example, low frequency to high frequency ratio (LF/HF ratio) reflects the distribution of power across different frequency bands, which is used as an indirect measure of sympathovagal balance, or the relational interplay between sympathetic and parasympathetic influences (Malik, 1996; Riganello et al., 2012). Time domain analyses of HRV reflect overall variability and short-term changes in cardiac output, reflecting sympathovagal balance (Sollers et al., 2007). Such measures have been investigated over short-term readings in response to situational changes; for example, social stress has been shown to have an acute impact on LF/HF ratio (Castaldo et al., 2015). Therefore, both chronic changes and the acute reactivity in HRV offer opportunities to measure ANS function that reflect the state of autonomic arousal (Hamilton & Alloy, 2016). Although much of the literature supports the use of HRV as a surrogate measure of autonomic activity, less research has assessed if changes in pupil activity reflect changes in autonomic activity.

The assessment of pupil function has been promoted as an alternative measure of ANS functioning (Reith et al., 2016), with commonly employed metrics including pupil diameter (Emelifeonwu et al., 2018). Increases in average pupil diameter throughout cognitive testing sessions serve as a reliable indicator of increased cognitive load (Mandrick et al., 2016; Wahn et al., 2016). Given that some cognitive tasks can influence autonomic arousal akin to a mild stressor (Horan et al., 2020), assessing pupillometry metrics during a task that manipulates states of demand and relaxation can offer valuable insights into ANS function. Correlations between mean pupil diameter and R-R intervals of HRV have been reported (Kaltsatou et al., 2011), prompting suggestions that HRV measures might be reliably estimated using pupil diameter metrics (Parnandi & Gutierrez-Osuna, 2013).

Reliability of HRV has generally been reported to be high within individuals across short time period measurements (<5 minutes) (Chen et al., 2020; Farah et al., 2016; Sookan & McKune, 2012). Although some authors have reported large random variations and moderate reliability in day-to-day readings of HRV (Dupuy et al., 2012; Sookan & McKune, 2012), the reliability of HRV metrics to detect reactivity across time and what constitutes a meaningful level of change has not been systematically investigated. Determining the minimum detectable change (MDC) is important to ensure that measurement error or natural fluctuations do not lead to inappropriate interpretations. Therefore, quantifying the day-to-day reliability of physiological measures reflecting ANS balance and reactivity is important to determine meaningful change from a typical state, which could identify individuals who are most affected by stressors and at greater risk of experiencing poor health outcomes (Brindle et al., 2014).

Combining HRV and pupillometry methods presents a promising approach for ANS evaluation by possibly capturing a wider scope of ANS function. The interaction between these metrics might enhance our understanding of an individual’s physiological state, potentially unveiling nuances that a single measurement approach might not reveal. However, little data exists to compare the reliability and reactivity of HRV and pupillometry metrics in response to changing controlled conditions. Therefore, the aim of this study was to investigate the day-to-day reliability and reactivity of HRV and pupillometry metrics in university students, under controlled conditions designed to perturbate the autonomic nervous system.

## Method

### Participants

Forty-one participants provided written consent in line with institutional ethics (HEC20077) to participate in this study. Using a recruitment script, Australian university students volunteered to participate in the study. Participants were included if they were current students and able to read English. Individuals with a history of loss of consciousness, psychiatric or neurological illness, dementia, head-injury, presence of current significant health-issues (including emotional disorders) or who used medications that might affect their ability to concentrate, or had health issues such as cardiac, mental, or thyroidal problems, physically fragility, or who were presently ill or unwell (cold, flu) were excluded from the study. Participants were then provided with written informed consent forms to participate in this study.

Using the COnsensus-based Standards for the selection of health Measurement Instruments (COSMIN) sample size tool (Mokkink et al., 2019) based on projected good average ICC (*r* = 0.8) with a 0.3 confidence interval (lower bound *r* = 0.65), a moderate amount of expected variance in scores and no systematic difference expected between our test–retest measures, a sample size of 30 participants was required to determine the test–retest reliability for this two-way mixed-effects model (absolute definition with average rater type) (Koo & Li, 2016). Additionally, 30 participants were sufficient to determine the reactivity of measures using a within-factor repeated measures ANOVA with a conservative small effect of ρ = 0.17, power set at 0.80, correlation of 0.7, and *p* = 0.05 (Version 3.1; G*Power, Kiel University, Germany).

### Experimental design

All participants repeated the test protocol at three timepoints in the same environmentally controlled (22 $^\circ{\rm C}$) quiet room without consuming fluids or food throughout testing. The first timepoint (*n* = 41) served to habituate participants with the protocol, and data from the second (Trial 1; *n* = 32) and third (Trial 2; *n* =30) timepoints (~1 week apart), were used to assess test–retest reliability. At each timepoint, a respiration band (Respiratory Belt Transducer; AD Instruments, USA) and a 5-lead Holter monitor (Medilog AR; Schiller, USA) were fitted according to manufacturer guidelines. Academic stress was assessed using the student form of the effort–reward imbalance (ERI) questionnaire, consisting of 14 items, measuring efforts, rewards, and overcommitment. Respondents rated each item on a four-point Likert scale ranging from 1 (“Strongly Disagree”) to 4 (“Strongly Agree”) (Wege et al., 2017). Psychological affect was measured with the depression, anxiety, stress scale- 21 (DASS-21), consisting of 21 items, with seven items each measuring levels of depression, anxiety, and stress. Respondents rated each item on a four-point Likert scale ranging from 0 (“Did not apply to me at all”) to 3 (“Applied to me very much or most of the time”) (Lovibond & Lovibond, 1995). They were then familiarized with the CONVIRT Virtual Reality (VR) environment (Horan et al., 2020) before completing three additional test phases in the VR environment: cognitive test battery and self-selected nature environment while undertaking 5 min of guided breathing at six breaths·min^−1^, and 5 min of spontaneous breathing. Recordings were stopped and participants removed the electrocardiogram (ECG) and breathing band before booking their next testing time, which was ~10 weeks after habituation. Trial 1 and Trial 2 were repeated at the same time of the day in the same lab room 1 week apart.

### Procedures

The ECG monitor (MediLog Holter; MediLog, USA) was applied to the participant’s chest via self-adhesive electrodes, using detailed instructions and a printed visual guide. Subsequently, the respiration band (LabCharts Respiration Monitor; ADInstruments, USA) was attached over clothing across the chest and connected to a data logger (PowerLab 26 Series; ADInstruments, USA) to calibrate the minimum expiration volume (> 0 mV) and maximum inspiration volume (< 100 mV). The HRV measure was then started, and participants completed the questionnaires. Participants completed a questionnaire that captured demographic information and assessed academic stress with the student form ERI (Wege et al., 2017), and psychological affect was measured with the DASS-21 (Lovibond & Lovibond, 1995). Participants were then equipped with the VR headset (HTC VIVE Pro Eye Tracking VR Head Mounted Display) and a handheld thumb-press button. After familiarization of the VR environment and eye-tracking calibration, four cognitive tests assessing attention, decision making and working memory were undertaken using the CONVIRT test battery (Horan et al., 2020). The CONVIRT testing environment includes eye-tracking to capture pupil metrics (HTC VIVE Pro Eye HMD has an eye tracking accuracy of 0–5–1.1 degrees with a refresh rate of 120 Hz) (VIVE^TM^ Australia, 2018) and provides a first-person perspective that the participant uses to navigate their way through a hospital setting (lux values from 60.6 to 70.1 cd/m^2^) where they responded with either a thumb press, visual gaze or both in response to specific commands. Participants were then shown an interface where they selected their preferred calm virtual environment (forest, beach, or lake). Each environment was matched for luminance (lux values: 70 cd/m^2^). After selection, pre-recorded verbal instructions on how to complete the guided breathing phase were provided within the environment, alongside an example visual. This visual showed a circle that progressively enlarged for 5 s with paired audio instructions to ‘breathe in’ followed by a progressive constriction of the circle paired with audio instructions to ‘breathe out’ for 5 s. Participants performed this behavior for 5 min to elicit a breathing rate of 6 breaths·min^−1^. The guided stimuli visual was then removed, and participants returned to free breathing in the same VR environment for a further 5 min.

### Measurement of HRV

Manufacturer’s software (Darwin V2; Medilog, Schiller AG, Switzerland) was used to process raw ECG data and determine R-to-R intervals. After removing non-sinus R-R intervals, the remaining N-N intervals were imported into a customized program developed in LabView (Version 2019; National Instruments, UK) to determine HRV outcomes in the time, frequency and non-linear domains in 1-min epochs according to standard methods (Malik, 1996). Time domain variables included standard deviation of the N-N intervals (SDNN), mean squared difference of successive N-N intervals (RMMSD), and the SDNN/RMMSD ratio. For frequency domain analyses, data were detrended and windowed using a Hanning window before fast Fourier transformation of 256 samples with 50% overlap to determine the power spectral density in LF bandwidth (0.04–0.15 Hz) and HF bandwidth (0.15–0.40 Hz) and LF/HF ratio as previously described (Kingsley et al., 2005). In addition, Poincaré SD1 (length of the transverse line of the Poincaré plot area), Poincaré SD2 (length of the longitudinal line of the Poincaré plot area), and the SD1/SD2 ratio were calculated because Poincaré analyses can be used to detect irregularities that may otherwise be difficult to determine with conventional time and frequency domain variables (Laitio et al., 2000).

### Methods of pupillometry

Pupil diameter, fluctuation, and asymmetry were measured using the eye-tracking feature of the HTC VIVE Pro Eye VR headset, which recorded changes in pupil diameter at a rate of 120 samples per second during the cognitive tasks. These measurements were then calculated across their respective testing phases. Pupil metrics included the mean pupil size, pupil fluctuation by calculating the standard deviation of pupil diameter data collected continuously within each phase, and pupil asymmetry by comparing the mean absolute difference between left and right pupils across each respective phase.

### Statistical analysis

All analyses were performed using the IBM SPSS Statistics computer software package (Version 29.0). Statistical significance was set at *p* < 0.05. Data were screened for normality using the Shapiro–Wilks statistic and breaches were identified across the majority of HRV and pupillometry metrics. Consequently, all HRV and pupillometry data were arcsine transformed prior to two-way repeated measures ANOVAs (within factors: trial and testing phase). Where the trial x phase interaction effects did not reach significance, the pattern of response was deemed to be consistent across trials and the main effects of trial and phase were consulted. Mauchly’s test of sphericity was consulted, and the Greenhouse–Geisser adjusted values were used when the assumption of sphericity was violated. Significant main effects of phase were followed up by pairwise comparisons with Bonferroni correction. Significant pairwise differences between adjacent phases were interpreted to demonstrate reactivity of the metric to changes in testing phases.

Intra-class correlations were used to measure the stability of individual data between trials, providing a measure of relative agreement according to guidelines with 95% confidence intervals (McGraw & Wong, 1996) after pooling with Fisher’s *Z* transformation (Silver & Dunlap, 1987). Two-way random effects of agreement on average scores were chosen to allow generalization of findings with ICC values interpreted as being “good” (0.75 to 0.90), “moderate” (0.5 to < 0.75) or “poor” (< 0.50) (Koo & Li, 2016). Ratio limits of agreement were used to quantify the absolute agreement between HRV and pupillometry metrics from Trial 1 to Trial 2 (day-to-day variation), where 95% ratio limit of agreement (95% RLOA) using the method for replicated data in pairs (Bland & Altman, 1986; Bland & Altman, 1999). Minimal detectable change (MDC) was calculated to identify the threshold value that can be considered as a change greater than the expected variation in the measurement on a day-to-day basis. The SEM was used to calculate the MDC with the equation MDC = SEM x *z*-score (95% CI), where the *z*-score equals 1.96 (McGraw & Wong, 1996).

## Results

Thirteen male and 17 female university students aged 18–35 years old (M = 21.8, SD = 4.0) with mean body mass of 68.8 kg (SD = 14.5), mean stature of 1.68 m (SD = 0.12) and mean body mass index 22.3 kg/m^2^ (SD = 4.2) completed all requirements and were included in analyses.

**Fig. 1:**
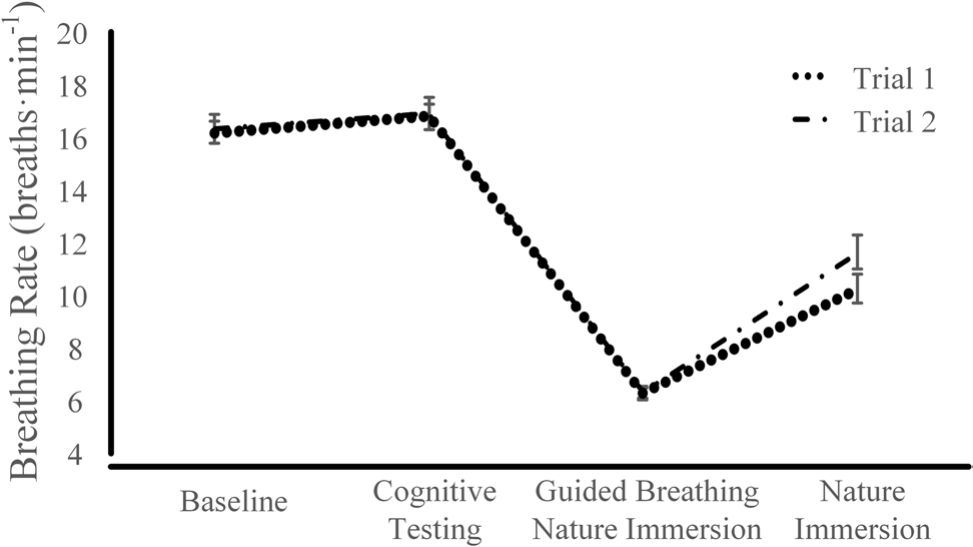
Mean (95% confidence interval) breathing rate between trials and across phase

Psychological affect did not significantly differ from Trial 1 to Trial 2, with no differences in academic stress (*t* = 1.12, *p* = 0.266), depression (*t* = 0.00, *p* = 0.999) or anxiety (*t* = 1.10, *p* = 0.283). Phasic changes in breathing rates are presented by trial in Fig. 1. Breathing rates were not statistically different between trials (trial effect: *F*_(1,29)_ = 2.38, *p* = 0.134, η^2^ = 0.08), but differed by intervention phase (phase effect: *F*_(3,87)_ = 209.68, *p* < 0.001, η^2^ = 0.88). Breathing rate did not significantly differ between baseline (16.3 breaths·min^−1^, 95% CI: 15.3 to 17.2 breaths·min^−1^) and cognitive testing (16.8 breaths·min^−1^, 95% CI: 15.8 to 17.9 breaths·min^−1^). Guided breathing, where participants were prompted to breathe at a rate of six breaths·min^−1^ with experiencing nature immersion, resulted in a reduction in breathing rate to 6.3 breaths·min^−1^ (95% CI: 5.8 to 6.8 breaths·min^−1^; *p* < 0.001) and breathing rate returned towards baseline breathing rates when guided breathing was withdrawn during the final phase with nature immersion (10.9 breaths·min^−1^, 95% CI: 10.0 to 11.9 breaths·min^−1^). Respiration depth (% of respiration range) did not differ significantly in pattern by trial (trial x phase interaction effect: *F*_(3,87)_ = 1.32, *p* = 0.272, η^2^ = 0.04), but differed by phase (*F*_(3,87)_ = 75.39, *p* < 0.001, η^2^ ≥ 0.72) with pairwise differences between cognitive testing to GBNI (Trail 1: MD: – 25%, 95% CI: – 33 to – 18%, *p* < 0.001, Trial 2: MD: – 29%, 95% CI: – 40 to – 18 %, *p* < 0.001) and GBNI to NI (Trial 1: MD: 20%, 95% CI: 13 to 27%, *p* < 0.001, Trial 2: MD: 25%, 95% CI: 14 to 37%, *p* < 0.001).

### Heart Rate Variability (HRV)

**Fig. 2:**
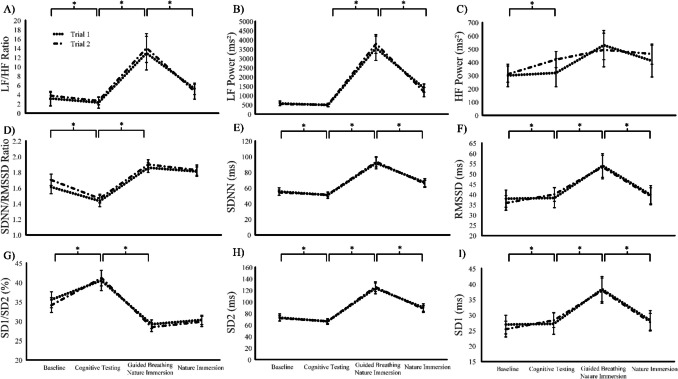
HRV scores (LF/HF ratio, SDNN/RMSSD ratio, SD1/SD2 ratio, LF power, SDNN, SD2, HF power, RMSSD and SD1) between trials and across phases

The pattern of response did not differ significantly across trials for all HRV metrics (trial x phase interaction effect: *F*_(3,87)_ ≤ 1.17, *p* ≥ 0.321, η^2^ ≤ 0.04) and no systematic between trial differences were observed for any HRV metrics (*F*_(1,29)_ ≤ 0.12, *p* ≥ 0.526, η^2^ ≤ 0.01; Fig. 2). Heart rate (HR) was also consistent across trials (trial x phase interaction effect: *F*_(3,87)_ = 2.92, *p* = 0.065, η^2^ = 0.09) with no systematic between trial differences (*F*_(1,29)_ = 0.88, *p* = 0.355, η^2^ = 0.03) and a significant difference between phases (*F*_(3,87)_ = 5.21, *p* = 0.005, η^2^ ≥ 0.15) from baseline to cognitive testing (MD: – 1.8 beats·min^−1^, 95% CI: – 1.3 to – 2.3 beats·min^−1^, *p* < 0.001, *d* = 0.94).

Day-to-day reliability and MDC for all HRV metrics are presented in Table 1. Relative reliability was good for all HRV metrics (ICC: 0.75 to 0.83). Absolute reliability had consistently low bias (1 to 6% for 78% of metrics) with variability ranging from SD1/SD2 ratio (RLOA: 0.90 to 1.05) to HF Power (RLOA: 0.67 to 1.20), being tighter for non-linear and time domain metrics in comparison to frequency domain metrics. The minimum detectable change in day-to-day values ranged from 22% (SDNN and SDNN/RMSSD ratio) to 73% of baseline values (HF power).

**Table 1:** Absolute reliability (bias and ratio limits of agreement), relative reliability (intra-class correlations) and minimal detectable change values for heart rate variability metrics

HRV metric	Mean bias (%)	RLOA (ratio)	ICC (95% CI)	MDC (metric unit)	MDC (% Baseline)
LF/HF ratio	1	0.78 to 1.26	0.76 (0.66 to 0.83)	1.85	54
LF Power (ms^2^)	15	0.69 to 1.11	0.83 (0.72 to 0.89)	183	32
HF Power (ms^2^)	12	0.67 to 1.20	0.77 (0.59 to 0.87)	225	73
SDNN/RMSSD ratio	2	0.95 to 1.09	0.76 (0.74 to 0.79)	0.38	22
SDNN (ms)	3	0.87 to 1.09	0.76 (0.66 to 0.83)	12	22
RMSSD (ms)	6	0.81 to 1.10	0.80 (0.69 to 0.87)	12	32
SD1/SD2 (%)	3	0.90 to 1.05	0.79 (0.76 to 0.81)	13	38
SD1 (ms)	6	0.81 to 1.10	0.80 (0.69 to 0.87)	8	31
SD2 (ms)	3	0.87 to 1.08	0.75 (0.65 to 0.82)	17	23

RLOA: ratio limits of agreement, ICC: intra-class correlation, CI: confidence interval, MDC: minimal detectable change, LF: low frequency, HF: high frequency, SDNN: standard deviation of normal-to-normal intervals, RMSSD: root mean square of successive differences, SD1: Poincare standard deviation 1, SD2: Poincare standard deviation 2

In the frequency domain, LF power, HF power and LF/HF ratio all differed by phase (*F*_(3,87)_ ≥ 6.96, *p* ≤ 0.002, η^2^ ≥ 0.19). LF/HF ratio was different for all phases of the protocol (Fig. 2A), with reactivity from baseline to cognitive testing (MD: – 1.0, 95% CI: – 0.6 to – 1.4, *p* < 0.001, *d* = 0.63), cognitive testing to guided breathing (MD: 11.1, 95% CI: 9.0 to 13.1, *p* < 0.001, *d* = 1.39) and guided breathing to nature immersion (MD: – 8.6, 95% CI: – 6.4 to – 10.8, *p* < 0.001, *d* = 0.99). LF power (Fig. 2B) increased from cognitive testing to guided breathing (MD: 3179 ms^2^, 95% CI: 3918 to 2442 ms^2^, *p* < 0.001, *d* = 1.11) and decreased from guided breathing to nature immersion (MD: – 2379 ms^2^, 95% CI: – 1679 to – 3078 ms^2^, *p* < 0.001, *d* = 0.88). HF power (Fig. 2C) increased from baseline to cognitive testing (MD: 64 ms^2^, 95% CI: 16 to 114 ms^2^, *p* = 0.002, *d* = 0.34).

In the time domain, SDNN, RMSSD and SDNN/RMSSD ratio were different between all phases (*F*_(3, 87)_ ≥ 22.09, *p* < 0.001, η^2^ ≥ 0.43) except for SDNN/RMSSD ratio from guided breathing to nature immersion (MD: –.06, 95% CI: – 0.02 to 0.14, *p* = 0.183 *d* = 0.17). SDNN/RMSSD ratio (Fig. 2D) decreased from baseline to cognitive testing (MD: – 0.21, 95% CI: – 0.15 to – 0.28, *p* < 0.001, *d* = 0.82) and increased to guided breathing (MD: 0.42, 95% CI : 0.34 to 0.49, *p* < 0.001, *d* = 1.34). SDNN (Fig. 2E) decreased from baseline to cognitive testing (MD: – 3.5 ms, 95% CI: – 1.3 to – 5.8 ms, *p* = 0.035, *d* = 0.40), increased to guided breathing (MD: 41.4 ms, 95% CI: −34.9 to – 47.9 ms, *p* < 0.001, *d* = 1.65) then decreased to nature immersion (MD: – 26.4 ms, 95% CI: – 19.8 to – 33.1 ms, *p* < 0.001, *d* = 1.03). RMSSD (Fig. 2F) increased from baseline to cognitive testing (MD: 2.2 ms, 95% CI: 0.4 to 4.1 ms, *p* = 0.004, *d* = 0.32), increased to guided breathing (MD: 14.6 ms, 95% CI: 10.0 to 19.1 ms, *p* < 0.001, *d* = 1.11) then reduced to nature immersion (MD: – 14.3 ms, 95% CI: – 9.6 to – 19.0 ms, *p* < 0.001, *d* = 0.78).

SD1/SD2 ratio, SD1 and SD2 all differed by phase (*F*_(3, 87)_ ≥ 22.07, *p* < 0.001, η^2^ ≥ 0.43; Fig. 2). SD1/SD2 ratio (Fig. 2G) increased from baseline to cognitive testing (MD: 0.06, 95% CI: 0.4 to 0.8, *p* < 0.001, *d* = 0.94) and decreased to guided breathing (MD: – 0.1, 95% CI: – 0.1 to – 0.1, *p* < 0.001, *d* = 1.16). SD2 (Fig. 2H) decreased from baseline to cognitive testing (MD: – 6.1 ms, 95% CI: – 2.9 to – 9.3 ms, *p* = 0.007, *d* = 0.51), increased to guided breathing (MD: 52.5 ms, 95% CI: 43.7 to 61.3 ms, *p* < 0.001, *d* = 1.61) then decreased to nature immersion (MD: – 36.5, 95% CI: – 27.0 to – 46.0 ms, *p* < 0.001, *d* = 1.03). SD1 (Fig. 2I) increased from baseline to cognitive testing (MD: 2.8 ms, 95% CI: 1.4 to 4.1 ms, *p* < 0.001, *d* = 0.54), increased to guided breathing (MD: 9.3 ms, 95% CI: 6.3 to 12.3 ms, *p* < 0.001, *d* = 0.83) and then decreased to nature immersion (MD: – 10.4 ms, 95% CI:– 6.8 to – 14.0 ms, *p* < 0.001, *d* = 0.78).

### Pupillometry

**Fig. 3:**
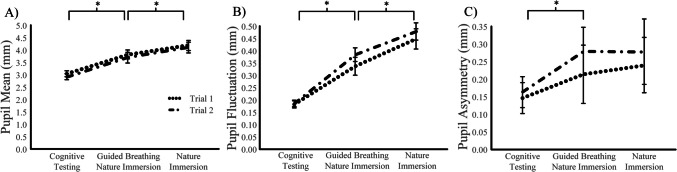
Pupillometry scores (pupil diameter, pupil fluctuation and pupil asymmetry) between trials across phases (mean and 95% confidence intervals)

The pattern of response was not significantly different across trials for all pupillometry metrics (trial x phase interaction effect: *F*_(1,29)_ ≤ 2.45, *p* ≥ 0.090, η^2^ ≤ 0.08). Systematic trial differences were observed for pupil diameter and pupil fluctuation metrics (trial effect: *F*_(1,29)_ ≥ 4.60, *p* ≤ 0.041, η^2^ ≥ 0.14) but not asymmetry (Trial effect: *F*_(1,29)_ =1.82, *p* = 0.188, η^2^ = 0.06). Each pupillometry metric differed between phase (Phase effect: *F*_(1,29)_ ≥ 12.15, *p* < 0.001, η^2^ ≥ 0.29) except for asymmetry from guided breathing to nature immersion (MD: 0.01 mm, 95% CI: – 0.02 to 0.05 mm, *p* = 1.00, *d* = 0.12; Fig. 3C).

Day-to-day reliability and MDC for all pupillometry metrics are presented in Table 2. Relative reliability was good for all pupillometry metrics (ICC: 0.66 to 0.87). Absolute reliability of diameter and fluctuation had consistently low bias (1 to 4%) with variability ranging from pupil fluctuation (RLOA: 1.03 to 1.19) to pupil diameter (RLOA: 0.92 to 0.99), being tighter than pupil asymmetry metrics (bias: 18%, RLOA: 0.88 to 2.59). The MDC change in day-to-day values ranged from 33% to 88% of baseline values for pupil diameter and asymmetry, respectively.

**Table 2:** Summary of reliability and minimal detectable change values for pupillometry measures

Measure	Mean bias (%)	RLOA ratio	ICC (95% CI)	MDC (mm)	Proportion of baseline mean (%)
Pupil diameter (mm)	2	0.92 to 0.99	0.87 (0.85 to 0.89)	0.22	33
Pupil fluctuation (mm)	4	1.03 to 1.19	0.67 (0.54 to 0.77)	0.29	64
Pupil asymmetry (mm)	18	0.88 to 2.59	0.66 (0.60 to 0.71)	1.47	88

RLOA: ratio limits of agreement, ICC: intra-class correlation, CI: confidence interval, MDC: minimal detectable change, SD: standard deviation

Pupil diameter increased from cognitive testing to guided breathing (MD: 0.43 mm, 95% CI: 0.01 to 0.08 mm, *p* < 0.001, *d* = 0.28) and then to nature immersion (MD: 0.75 mm, 95% CI: 0.64 to 0.88 mm, *p* < 0.001, *d* = 1.61; Fig. 3A). Pupil fluctuation increased from cognitive testing to guided breathing (MD: 0.27 mm, 95% CI: 0.23 to 0.30, *p* < 0.001,* d* = 1.91) and then to nature immersion (MD: 0.16 mm, 95% CI: 0.11 to 0.21 mm, *p* < 0.001,* d* = 0.81; Fig. 3B). Pupil asymmetry increased from cognitive testing to guided breathing (MD: 0.10 mm, 95% CI: 0.05 to 0.14 mm, *p* < 0.001,* d* = 0.57).

## Discussion

The results of this study confirm strong day-to-day relative reliability for HRV and pupillometry measurement, taken 1 week apart, with academic stress and affect remaining stable, indicating good stability of measurement over time. HRV metrics of sympathovagal balance and pupillometry displayed discernible reactivity throughout the test phases. These findings demonstrate the effectiveness of both cognitive testing and paced breathing to modulate physiological responsiveness, suggesting that the current protocol manipulates elements of psychological demand and relaxation that is suitable to detect physiological reactivity using HRV and pupillometry metrics.

### Day-to-day reliability of heart rate variability and pupillometry

When investigating reliability, the distinction between relative and absolute reliability is important (Maestri et al., 2010; Weir, 2005). Relative reliability considers the extent to which individuals maintain their within-sample rank across repeated measurements (Sole et al., 2007; Watson, 2004; Weir, 2005). In line with the current findings, where ICCs ranged from 0.75 to 0.83 for HRV metrics, HRV measures have previously been reported to demonstrate good relative reliability across short time intervals (Bertsch et al., 2012; Cipryan & Litschmannova, 2013, 2014; Kobayashi, 2009; Maestri et al., 2010; Pinna et al., 2007). The relative reliability of pupillometry measures was moderate for pupil fluctuations and asymmetry, and good for pupil diameter (Couret et al., 2016; Farah et al., 2016).

Absolute reliability considers the variation in repeated measures for an individual, regardless of their relative position (Atkinson & Nevill, 1998). Mean bias was low (≤ 6%) for the majority of HRV metrics, but the range in RLOA was variable with the tightest being SD2 and the broadest being HF power. Although the absolute reliability of HRV reactivity across timepoints has received little attention, these results extend previous finding that individual variations in HRV have been shown to be high (Dupuy et al., 2012; Sookan & McKune, 2012) with large variability being reported for absolute reliability (Cipryan & Litschmannova, 2013, 2014; Maestri et al., 2010; Pinna et al., 2007).

### Reactivity of heart rate variability and pupillometry to changes in cognitive stimulation and breathing

The breathing patterns of participants in response to the test protocol were consistent across trials, with similar breathing rate and depth during baseline and cognitive testing (~16 breaths·min^−1^), decreasing to ~6 breaths·min^−1^ during paced breathing and then returning towards baseline values when paced breathing was withdrawn. The high reactivity that was observed in the HRV measures between guided breathing with nature immersion to spontaneous breathing with nature immersion can therefore be attributed to respiratory modulation. This response was expected and in accordance with the established association of respiration-driven changes in HRV occurring through vagally mediated respiratory sinus arrythmia (Kromenacker et al., 2018; Miu et al., 2009; Pagani et al., 1984). Stability in the breathing rate and depth across baseline and cognitive testing demonstrates that changes in physiological reactivity from baseline to cognitive testing were not driven by respiratory modulation. Furthermore, heart rate displayed strong reliability between trials and only a small change was identified from baseline to cognitive testing (MD: – 1.8 beats·min^−1^). The consistency of heart rate across phases demonstrates that it was unlikely to systematically influence the HRV results in this study.

HRV measures of sympathovagal balance (LF/HF ratio, SDNN/RMSSD ratio and SD1/SD2) all showed change from baseline to cognitive testing, with SD1/SD2 displaying the expected reciprocal pattern of response when compared with LF/HF ratio and SDNN/RMSSD. This similarity in reactivity aligns with the known correlation between LF/HF ratio and SD1/SD2 (Guzik et al., 2007; Zerr et al., 2015). The change from baseline to cognitive testing, while the virtual environment and respiration remained consistent, demonstrates sensitivity of these HRV measures to changes in cognitive load as the cognitive distractor influenced parasympathetic balance matching previous findings (Manser et al., 2021). The finding that breathing at 6 breaths·min^−1^ increased power predominantly in the traditional LF bandwidth is consistent with the known influence of slow-paced breathing on autonomic regulation (Lehrer, 2007), and high reactivity in response to guided breathing aligns with the influence of respiratory modulation of the ANS. Furthermore, the reversal in direction in these measures of sympathovagal balance after baseline and then cognitive testing strengthens these findings by discounting systematic temporal influences, such as habituation.

LF power, SDNN and SD2 are traditionally associated with SNS activity but also include PNS activity (Shaffer et al., 2014). As expected, due to the high correlations between LF power, SDNN (Umetani et al., 1998), and SD2 (Brennan et al., 2002), the results across these metrics were consistent. Similar to the current study, LF power calculated while sitting upright has been shown to reflect a higher contribution from PNS activity (Berntson et al., 1997; Eckberg, 1983). The seated position might explain the similar trend observed to sympathovagally mediated metrics, with both SNS and PNS contributions being detected.

HF power, RMSSD, and SD1 estimate PNS activity and indicate vagally mediated changes in HRV (Kleiger et al., 2005; Shaffer et al., 2014). HF power is highly correlated with RMSSD (Kleiger et al., 1987) while RMSSD and SD1 provide identical measures (Ciccone et al., 2017). RMSSD and SD1 displayed changes across all phases, with the greatest change occurring during guided breathing nature immersion. These metrics all increased from baseline to cognitive testing. Therefore, detection of change due to a mild cognitive stressor might include SNS activation, which is less influential in these metrics. HF power only showed a change from baseline to cognitive testing. HF power displayed weaker reliability and less reactivity than the other similar metrics, suggesting HF power is a less reliable measure of PNS reactivity during this protocol. Conversely, HRV metrics with PNS and SNS components were more sensitive to reactivity throughout the phases.

With the exception of pupil asymmetry, pupillometry metrics all showed increases from cognitive testing to guided breathing nature immersion to nature immersion. No measure of pupillometry was taken at the baseline phase because the luminance in this static environment was not consistent with the luminance of the testing environment. During the assessed phases, luminance was stable (lux values ranged from 70 cd/m^2^ in guided breathing to 61 cd/m^2^ in nature immersion). Systemic differences between trials for pupil diameter and fluctuation might be explained by differences in baseline levels as trends remained constant across phases. Increases from cognitive testing to guided breathing nature immersion matched HRV metrics, although the continued increase from guided breathing nature immersion to nature immersion was distinct for pupillometry metrics. It has been established that inhalation and expiration affect pupil diameter (Borgdorff, 1975; Häbler et al., 1994), but breathing rate and deep breathing at six breaths·min^−1^ do not (Debnath et al., 2021; Schaefer et al., 2023). The divergent mechanisms underlying pupillometry and HRV across cognitive testing and paced breathing extend on other findings indicating an alternative control mechanism in the cognitive state of task engagement, through which respiratory driven autonomic control is superseded (Nakamura et al., 2019). Contrary to expectations, each pupil metric increased across phases, which matched the order of most cognitively demanding (cognitive testing) to least cognitively demanding (nature immersion). As luminance was controlled, and breathing rate is unlikely to have influenced pupillary measures, the increases across phases might not be explained by cognitive load, but by changes in psychological affect, a known factor impacting pupillometry function (Carvalho et al., 2015; Peinkhofer et al., 2019; Thomas et al., 2021). Previous findings demonstrate that both meditation and virtual nature immersion are associated with increases in positive affect (Frost et al., 2022), and such changes in affect are associated with modulation of pupil diameter (Tichon et al., 2014). Although state affect was not measured in the current study, it likely improved in line with immersion in a calming environment of the participant's choice. Therefore, even though pupillometry and HRV are autonomically influenced, these findings indicate that differential influences exist between these measures.

### Minimal detectable change

The MDC values, when presented as percentage change from baseline, ranged from 22% for SDNN/RMSSD ratio to 88% for pupil asymmetry. On an individual basis, MDC values have been applied in practical settings as the amount of change in repeated measurements that can be interpreted as exceeding normal day-to-day variability. The relatively large MDC values in this study are expected for physiological measures which are known to fluctuate regularly, and explain the difficulty in using these indices to detect systematic changes from one time point to another at an individual level.

### Limitations

Several confounds inherent in HRV and pupillometry measurement need consideration. HRV is known to be sensitive to various conditions, including posture, postural changes, and ambient temperature. Standardized testing conditions were implemented to address these nuisance factors, with participants remaining seated throughout testing in a stable laboratory condition of 22 $^\circ{\rm C}$. Pupillometry measures are influenced by light and the CONVIRT system ensured luminance values were stable during testing. To preserve statistical power, other factors such as fitness and BMI were not included as covariates in analyses but the impact of between-person variability is likely minimal given the within-subjects design employed. Respiration belt accuracy was likely enhanced by a standardized protocol where participants were fitted before HRV recordings to ensure accurate sensitivity and were monitored throughout testing. Finally, although the sample size in the present study provided adequate statistical power for our analyses, the generalizability of our findings would be improved with a larger and more diverse sample.

## Conclusion

Across all metrics, relative day-to-day reliability was high with minimal systematic differences between repeated measures, meaning that these measures are likely to be useful to identify differences in means between groups. Absolute reliability was characterized by relatively wide RLOA values and MDC values were high, suggesting that these metrics are less likely to be useful in detecting change at the individual level. The intervention protocol used in this study elicited physiological reactivity in a repeatable fashion over time, and HRV and pupillometry metrics offer promising measures to assess the reactivity of individuals to changes in psychological activities and states. Protocols that provide controlled conditions with varying psychological activities (e.g., demands and relaxation) should be considered when researchers wish to monitor physiological reactivity of the ANS using HRV and pupillometry measurements.

## Data Availability

The data for the project are available at https://osf.io/e695b/
